# Effectively communicating with local policymakers: a randomized trial of policy brief dissemination to address obesity

**DOI:** 10.3389/fpubh.2024.1246897

**Published:** 2024-03-08

**Authors:** Elizabeth A. Dodson, Renee G. Parks, Rebekah R. Jacob, Ruopeng An, Amy A. Eyler, Nathan Lee, Alexandra B. Morshed, Mary C. Politi, Rachel G. Tabak, Yan Yan, Ross C. Brownson

**Affiliations:** ^1^Prevention Research Center, Brown School at Washington University in St. Louis, St. Louis, MO, United States; ^2^Brown School, Washington University in St. Louis, St. Louis, MO, United States; ^3^CivicPulse, Portland, OR, United States; ^4^Rollins School of Public Health, Emory University, Atlanta, GA, United States; ^5^Division of Public Health Sciences, Department of Surgery, Washington University School of Medicine, St. Louis, MO, United States; ^6^Division of Public Health Sciences, Department of Surgery, Division of Biostatistics, Washington University School of Medicine, St. Louis, MO, United States; ^7^Department of Surgery, Division of Public Health Sciences, and Alvin J. Siteman Cancer Center, Washington University School of Medicine, St. Louis, MO, United States

**Keywords:** communication, dissemination, evidence-based policy, local policy, obesity policy

## Abstract

**Introduction:**

Evidence-based policies are a powerful tool for impacting health and addressing obesity. Effectively communicating evidence to policymakers is critical to ensure evidence is incorporated into policies. While all public health is local, limited knowledge exists regarding effective approaches for improving local policymakers' uptake of evidence-based policies.

**Methods:**

Local policymakers were randomized to view one of four versions of a policy brief (usual care, narrative, risk-framing, and narrative/risk-framing combination). They then answered a brief survey including questions about their impressions of the brief, their likelihood of using it, and how they determine legislative priorities.

**Results:**

Responses from 331 participants indicated that a majority rated local data (92%), constituent needs/opinions (92%), and cost-effectiveness data (89%) as important or very important in determining what issues they work on. The majority of respondents agreed or strongly agreed that briefs were understandable (87%), believable (77%), and held their attention (74%) with no brief version rated significantly higher than the others. Across the four types of briefs, 42% indicated they were likely to use the brief. Logistic regression models showed that those indicating that local data were important in determining what they work on were over seven times more likely to use the policy brief than those indicating that local data were less important in determining what they work on (aOR = 7.39, 95% CI = 1.86,52.57).

**Discussion:**

Among local policymakers in this study there was no dominant format or type of policy brief; all brief types were rated similarly highly. This highlights the importance of carefully crafting clear, succinct, credible, and understandable policy briefs, using different formats depending on communication objectives. Participants indicated a strong preference for receiving materials incorporating local data. To ensure maximum effect, every effort should be made to include data relevant to a policymaker's local area in policy communications.

## Introduction

Obesity affects over one in three U.S. adults and one in five children with estimated annual medical costs reaching nearly $173 billion (1). Evidence-based policies (EBPs) to address obesity prevention exist, but are not systematically applied (2, 3). EBPs have historically had significant influences on public health (e.g., use of seat belts, protection of employees in the workplace) (4). Thus, policy is a powerful tool for improving population health, and policymakers are in positions to enact policies with potential to significantly impact health and reduce obesity (5). Therefore, effectively communicating evidence to policymakers is critical for improving the likelihood that it will be incorporated into policies (5, 6).

There are myriad barriers to effectively communicating evidence-based public health data to policymakers (7, 8). Policymakers and researchers often have conflicting decision-making processes, different timelines, and varying levels of uncertainty in information (2). Further, policymakers function in a world of information overload, receiving hundreds of pieces of information from varied sources every day. In one study, policymakers reported only reading for detail 27% of what they receive and never getting to 35% (9). Finally, policymakers may struggle with finding the data they need when they need it (10). Previous research addresses how policymakers prefer to receive information and what types they seek. These preferences commonly include local data showing economic costs presented in a brief format that is timely and easy to understand (10, 11). Up to 61% of state legislators report that they prioritize unbiased and understandable research (12). Other studies have concluded that there is no “one size fits all” approach to policymaker communication; rather, messages should be tailored to the type of policymaker or utilize audience segmentation (3, 12, 13).

While previous studies present policymaker preferences at the federal and state level, limited knowledge exists regarding effective approaches for the uptake of research-tested, policy interventions among local policymakers (14). All public health is local and tremendous potential exists for addressing obesity through local policies that encourage healthy eating and physical activity (15–18). For example, initiatives to improve access to safe places for physical activity may be accomplished through local zoning and land use ordinances (19, 20). Further, local policymakers are commonly attuned to constituent opinions and data about their local jurisdictions (21). Thus, ensuring that local policymakers have access to information about EBPs presented in relevant and easily-digestible ways is crucial to improving the uptake of EBPs in local policy and ultimately to addressing the burden of obesity.

Accordingly, to effectively translate relevant research to local policy, the purpose of this study was to test a set of approaches for the translation of research to local policymakers.

## Materials and methods

This trial was part of a larger study designed to develop and disseminate approaches to increase the implementation of EBPs to reduce obesity disparities and promote health equity, focusing on the uptake of effective local-level policies. One aim of this study was to test a set of approaches for translation of research about obesity EBPs among local policymakers. To this end, we conducted a randomized trial of four types of policy briefs with local policymakers. This allowed for the comparison of different policy brief formats, the examination of various factors influencing issues local policymakers choose to work on, and the contribution of these factors to the likelihood that local policymakers would use the policy briefs.

### Policy brief development and content

Four versions of a policy brief were created: usual care, risk framing, narrative, and risk framing plus narrative (mixed). These types were selected based on previous research indicating that narrative forms of communication (compared to a traditional data-oriented presentation of information) can improve understanding of complex information (10, 22, 23). Further, risk framing, which communicates risks using more easily-understandable means (e.g., frequencies, percentages, graphs) is often used in medical decision-making, and may be a promising approach for clarifying policy options, such as the risks and benefits of various interventions, in a local policy context (24, 25). The topic of each brief, zoning and development regulations, was the same for each type. This topic was chosen based on its relevance at the local government level as well as findings from prior research conducted as part of the more extensive study. This research included qualitative interviews with municipal officials and sought, in part, to understand policymaking influences at the local level. Most of the health-related policy action examples mentioned in these interviews are impacted by zoning and development regulations (e.g., food and physical activity environments, housing affordability); thus, the topic was selected for the policy brief randomized trial.

With support from health communication and decision science experts, the four types of policy briefs were developed and refined. The usual care briefs included traditional content for health experts; however, the text was condensed and revised to include plain language and recommended design principles for summarizing scientific language (e.g., use of white space, use of pictures/icons to convey meaning, etc.). The narrative brief included stories presenting protagonists with similar situations but in contrasting settings (one in an area zoned for single-family residences and one zoned for mixed uses) and the impact on daily life. The risk-framing briefs used principles from decision sciences to frame data in meaningful and accessible ways. These briefs were designed to relate specific risk data to local policymakers using social math and meaningful visuals. The risk framing plus narrative briefs combined both narrative and risk framing communication elements. There were two consistent sections across brief versions (for content and look/design): “What can local governments do” (i.e., succinct policy actions), and “Impact” (i.e., briefly stated benefits to the community). All four brief types are available here: https://prcstl.wustl.edu/items/prc-core-research-project/.

Formative testing of the brief versions was conducted with a Community Advisory Board, which is comprised of local policymakers, public health practitioners at the local and state levels, and representatives of public health advocacy organizations. This testing was designed to ensure distinction in policy brief versions. All board members who tested the briefs correctly identified the versions based on a short description.

### Survey measures

The research team developed, edited, and tested a brief survey to accompany the policy briefs. The survey design was pilot tested to assess respondent completion and address concerns about the cognitive load of reading a policy brief and responding to survey items. Two survey designs were tested; both performed similarly well for response of survey items, including open-ended text items, and completion rates. The research team determined to administer the original design, including the four policy briefs described above. Measures were based on previous research (3, 26). The survey, created with Qualtrics (27), included 18 items, three of which were open-ended, and was designed to be completed in 10 min or less.

#### Covariates

Policymakers were first asked how important a list of factors is in determining issues they work on, using a five-point Likert scale ranging from unimportant to very important. These factors included personal interest, data on the impact in their local area or community, constituent needs or opinions, recommendations of local organizations, evidence of scientific effectiveness, and availability of cost-effectiveness or economic analysis. In addition, participants were asked to rate the level of importance of issues that affect their community, including crime and violence, economy, education, environment, mental health, and physical health.

Next, through simple random allocation (programmed within the Qualtrics survey), participants were presented one policy brief version and asked a series of questions representing various domains. Each question included response options on a five-point Likert scale ranging from strongly disagree to strongly agree. To assess understanding, participants were asked if the information in the policy brief was easy to understand, held their attention, and affected them emotionally. To assess credibility, participants were asked if the information in the policy brief was believable, accurate, and whether it provided a strong reason for local governments to implement zoning and development regulations to address obesity and promote health. Participants were also asked whether they were likely to use the information in the policy brief.

Participants were asked about their political ideology on social and fiscal issues, with response options ranging from extremely conservative to extremely liberal on a seven-point scale. The survey also asked demographic questions (e.g., age, gender, political party). Finally, participants were asked if they have any educational background or experience in land use, planning, or physical design of public spaces, with response options including: a great deal, a fair amount, a little bit, or none. The full survey instrument is available at the link above.

### Sample selection and recruitment

The research sample was randomly drawn from a population of elected policymakers representing local U.S. governments with over 1,000 residents, including those at the county (county executives and commissioners), municipal (mayors and councilmembers), and township levels. To select the sample and administer the survey, the research team collaborated with CivicPulse, a non-profit organization focused on producing knowledge of and for local governments through national surveys of local officials. The sample was drawn from their national panel of local government leaders (28). The Institutional Review Board of Washington University in St. Louis approved this study as exempt research (#202110030).

### Data collection

CivicPulse conducted data collection between January and March 2022. Respondents were invited via email to take the survey. Three email attempts were made. Consent to participate in the survey was implied when participants followed the survey link.

When participants clicked on the link to complete the survey, they found the policy brief embedded as a graphic within the online survey. This was done for simplicity and to minimize attrition. Incentives were not offered to participants; however, as a benefit to their panel of local government officials, CivicPulse provides a summary of research findings via posts on their website and emailed newsletters, within a few months of survey completion.

### Data analysis

Frequencies were run for all categorical variables to examine differences across the study group (policy brief shown), and differences in outcomes were assessed using Pearson's Chi-squared test and ANOVA models, as appropriate. Political ideology responses were collapsed to form three groups: conservative/slightly conservative, moderate, and liberal/slightly liberal. The variables describing the importance of various factors in determining what issues a policymaker works on were stratified by political party. Response options for these questions were collapsed to create the following categories: very important/important, moderately important, and slightly important/not important.

Response options for each variable used to describe policymakers' ratings of the briefs, in terms of understandability and credibility, were combined to create two categories: Agree (mostly agree/strongly agree) and Else (undecided, mostly disagree, strongly disagree). We conducted a factor analysis of six policy brief ratings (understandable, attention held, emotion evoked, believable, accurate, strong reasoning). Factor analysis was used as a dimension reduction method to identify similar dimensions of complex sets of variables and aid in the interpretability of relationships to our dependent variable without a significant loss of degrees of freedom and overfitting models (29). A one-factor solution was reached using orthogonal (varimax) rotation and Thompson's estimator for regression score calculations. The proportion of variance explained with the one-factor solution was 0.47. The resulting variable (understandability/credibility factor score) was used in logistic regression models described below.

Logistic regression models were used to analyze the effects of various factors on the likelihood of brief use, the primary dependent variable (strongly agree/agree = yes, and undecided/disagree/strongly disagree = no). The first or null model included a randomization group (type of brief shown) with the usual care brief as the reference. The second model added two items found to significantly improve model deviance from factors policymakers indicated were important/very important in determining what issues to work on (data on impact in local area/community and evidence of scientific effectiveness). The third model included the understandability/credibility factor score, the one-factor representative of the six variables in which participants reflected on the briefs (i.e., understandable, held my attention, affected me emotionally, believable, accurate, and whether the brief provided a strong reason for governments to implement zoning regulations). All models applied probability weighting based on a post-stratification raking procedure using Census and presidential vote share variables (30). Odds ratios and 95% confidence estimates were calculated for each model. All data cleaning and analysis were performed in R version 4.1.2 (31).

## Results

The total number of policymakers invited to participate was 7,950, of whom 331 finished the survey, resulting in a completion rate of 4.5%. This response rate is similar to that of other nationally representative surveys of local public officials (32–34). The median time spent viewing the brief and answering survey questions was 8.4 min. A majority of respondents was non-Hispanic white (84%) men (68%) who hold college or graduate degrees (72%). One-third of the sample was between 52–66 years old, while nearly 48% were over age 67. The average time spent in participants' current positions was 9 years. The sample was relatively evenly distributed in terms of political party, experience with land use, and social ideology. Sixty-three percent worked at the municipal level, 20% at the township level, and 16% at the county level (Table 1).

**Table 1:** Demographic characteristics of local policymaker study participants, *N* = 331.

	**Usual (*N =* 82)**	**Framing (*N =* 82)**	**Narrative (*N =* 83)**	**Mixed (*N =* 84)**	**Total (*N =* 331)**	***p*-value**
**Age category**						0.587^a^
22–51	11 (13.8%)	15 (19.7%)	20 (24.4%)	18 (21.7%)	64 (19.9%)	
52–66	26 (32.5%)	29 (38.2%)	24 (29.3%)	26 (31.3%)	105 (32.7%)	
67+	43 (53.8%)	32 (42.1%)	38 (46.3%)	39 (47.0%)	152 (47.4%)	
**Gender**						0.710^b^
Female	29 (35.4%)	26 (32.5%)	24 (29.6%)	25 (30.1%)	104 (31.9%)	
Male	53 (64.6%)	54 (67.5%)	56 (69.1%)	58 (69.9%)	221 (67.8%)	
Prefer to self-describe	0 (0.0%)	0 (0.0%)	1 (1.2%)	0 (0.0%)	1 (0.3%)	
**Highest education attained**						0.577^a^
Graduate degree	31 (37.8%)	26 (32.1%)	24 (28.9%)	32 (38.1%)	113 (34.2%)	
College graduate or some graduate school	32 (39.0%)	25 (30.9%)	33 (39.8%)	34 (40.5%)	124 (37.6%)	
Some college or technical/trade school	14 (17.1%)	23 (28.4%)	20 (24.1%)	13 (15.5%)	70 (21.2%)	
High school or less	5 (6.1%)	7 (8.6%)	6 (7.2%)	5 (6.0%)	23 (7.0%)	
**Race and ethnicity**						0.519^a^
Non-hispanic white	71 (86.6%)	70 (86.4%)	64 (79.0%)	69 (84.1%)	274 (84.0%)	
Non-white or hispanic	11 (13.4%)	11 (13.6%)	17 (21.0%)	13 (15.9%)	52 (16.0%)	
**Years in current position**						0.500^b^
Mean (CI)	8.34 (6.77, 9.91)	10.05 (8.20, 11.90)	8.45 (6.69, 10.21)	9.00 (7.19, 10.81)	8.95 (8.09, 9.81)	
**Land use experience**						0.688^a^
A fair amount/a great deal	27 (32.9%)	27 (34.2%)	25 (30.1%)	33 (39.8%)	112 (34.3%)	
A little bit	23 (28.0%)	19 (24.1%)	27 (32.5%)	25 (30.1%)	94 (28.7%)	
None	32 (39.0%)	33 (41.8%)	31 (37.3%)	25 (30.1%)	121 (37.0%)	
**Political party**						0.386^a^
Democrat	25 (31.2%)	24 (30.4%)	20 (24.7%)	26 (31.0%)	95 (29.3%)	
Republican	29 (36.2%)	38 (48.1%)	35 (43.2%)	32 (38.1%)	134 (41.4%)	
Independent	24 (30.0%)	16 (20.3%)	22 (27.2%)	26 (31.0%)	88 (27.2%)	
Other party	2 (2.5%)	1 (1.3%)	4 (4.9%)	0 (0.0%)	7 (2.2%)	
**Social position**						0.122^a^
Conservative/slightly conservative	24 (29.3%)	38 (47.5%)	37 (45.1%)	37 (44.0%)	136 (41.5%)	
Moderate	33 (40.2%)	20 (25.0%)	26 (31.7%)	20 (23.8%)	99 (30.2%)	
Liberal/slightly liberal	25 (30.5%)	22 (27.5%)	19 (23.2%)	27 (32.1%)	93 (28.4%)	
**Fiscal position**						0.832^a^
Conservative/slightly conservative	51 (62.2%)	49 (61.2%)	50 (61.0%)	58 (69.0%)	208 (63.4%)	
Moderate	25 (30.5%)	25 (31.2%)	25 (30.5%)	18 (21.4%)	93 (28.4%)	
Liberal/slightly liberal	6 (7.3%)	6 (7.5%)	7 (8.5%)	8 (9.5%)	27 (8.2%)	
**Government level of respondent**						0.007^a^
County	14 (17.1%)	6 (7.3%)	18 (21.7%)	16 (19.0%)	54 (16.3%)	
Municipality	60 (73.2%)	59 (72.0%)	47 (56.6%)	44 (52.4%)	210 (63.4%)	
Township	8 (9.8%)	17 (20.7%)	18 (21.7%)	24 (28.6%)	67 (20.2%)	

^a^Pearson's Chi-squared test.

^b^Linear Model ANOVA.

When asked to rate the importance of various factors in determining what issues they worked on, 92.1% of participants rated data on impact in their local area or community as important or very important, as well as constituent needs or opinions (92.1%) and data on cost-effectiveness (88.9%) (Table 2). Further, evidence of scientific effectiveness was rated as important/very important by 81.3% of participants. By contrast, only 43.6% of participants reported that personal interest was important or very important in determining what issues they worked on.

**Table 2:** Relative importance of various factors in determining what issues local policymakers work on.

	**Democrat (*N =* 95)**	**Republican (*N =* 134)**	**Independent (*N =* 88)**	**Total (*N =* 317)**	***p*-value**
**Personal interest**					0.400^a^
Important/very important	41 (43.6%)	53 (39.8%)	43 (49.4%)	137 (43.6%)	
Moderately important	30 (31.9%)	36 (27.1%)	24 (27.6%)	90 (28.7%)	
Not/slightly important	23 (24.5%)	44 (33.1%)	20 (23.0%)	87 (27.7%)	
**Data on impact in local area/community**					0.559^a^
Important/very important	89 (94.7%)	121 (91.0%)	80 (90.9%)	290 (92.1%)	
Moderately important	4 (4.3%)	10 (7.5%)	8 (9.1%)	22 (7.0%)	
Not/slightly important	1 (1.1%)	2 (1.5%)	0 (0.0%)	3 (1.0%)	
**Constituent needs or opinions**					0.187^a^
Important/very important	84 (89.4%)	123 (91.8%)	84 (95.5%)	291 (92.1%)	
Moderately important	8 (8.5%)	11 (8.2%)	4 (4.5%)	23 (7.3%)	
Not/slightly important	2 (2.1%)	0 (0.0%)	0 (0.0%)	2 (0.6%)	
**Recommendations of local organizations**					0.466^a^
Important/very important	64 (68.1%)	94 (70.1%)	52 (59.1%)	210 (66.5%)	
Moderately important	26 (27.7%)	32 (23.9%)	30 (34.1%)	88 (27.8%)	
Not/slightly important	4 (4.3%)	8 (6.0%)	6 (6.8%)	18 (5.7%)	
**Evidence of scientific effectiveness**					0.184^a^
Important/very important	82 (87.2%)	105 (78.4%)	70 (79.5%)	257 (81.3%)	
Moderately important	11 (11.7%)	21 (15.7%)	16 (18.2%)	48 (15.2%)	
Not/slightly important	1 (1.1%)	8 (6.0%)	2 (2.3%)	11 (3.5%)	
**Cost effectiveness or economic analysis**					0.035^a^
Important/very important	79 (84.0%)	124 (92.5%)	78 (88.6%)	281 (88.9%)	
Moderately important	15 (16.0%)	9 (6.7%)	7 (8.0%)	31 (9.8%)	
Not/slightly important	0 (0.0%)	1 (0.7%)	3 (3.4%)	(1.3%)	

^a^Pearson's Chi-squared test.

When participants were asked to rate the policy briefs on a range of factors, the majority agreed or strongly agreed that the briefs were easy to understand (86.9%), believable (76.5%), accurate (52.7%), and held their attention (74.4%). About one-third of participants reported that the briefs affected them emotionally (32.2%). When asked if they were likely to use the information in the policy briefs, 41.6% agreed or strongly agreed that they were. These responses were primarily consistent across the various policy brief types (Table 3). Finally, 46% of respondents agreed or strongly agreed that the brief provided a strong reason for local governments to implement zoning and development regulations to address obesity and promote health. Responses to this item varied across types of policy brief. Those receiving the usual care brief were significantly more likely to agree or strongly agree with this item (58.5%), and those receiving the narrative brief were significantly less likely to agree or strongly agree with the item (31.7%; *p* = 0.004), as shown in Table 3.

**Table 3:** Local policymakers' ratings of characteristics of policy briefs, by type of brief shown.

	**Usual (*N =* 82)**	**Framing (*N =* 82)**	**Narrative (*N =* 83)**	**Mixed (*N =* 84)**	**Total (*N =* 331)**	***p*-value**
**Understandability**						
**Easy to understand**						0.202^a^
Agree	75 (91.5%)	70 (86.4%)	73 (89.0%)	67 (80.7%)	285 (86.9%)	
Else	7 (8.5%)	11 (13.6%)	9 (11.0%)	16 (19.3%)	43 (13.1%)	
**Held attention**						0.578^a^
Agree	60 (73.2%)	65 (80.2%)	59 (72.0%)	60 (72.3%)	244 (74.4%)	
Else	22 (26.8%)	16 (19.8%)	23 (28.0%)	23 (27.7%)	84 (25.6%)	
**Affected emotionally**						0.228^a^
Agree	21 (25.6%)	23 (28.4%)	31 (38.8%)	30 (36.1%)	105 (32.2%)	
Else	61 (74.4%)	58 (71.6%)	49 (61.2%)	53 (63.9%)	221 (67.8%)	
**Credibility**						
**Believable**						0.782^a^
Agree	66 (80.5%)	62 (76.5%)	61 (74.4%)	62 (74.7%)	251 (76.5%)	
Else	16 (19.5%)	19 (23.5%)	21 (25.6%)	21 (25.3%)	77 (23.5%)	
**Accurate**						0.254^a^
Agree	51 (62.2%)	39 (48.1%)	42 (51.2%)	41 (49.4%)	173 (52.7%)	
Else	31 (37.8%)	42 (51.9%)	40 (48.8%)	42 (50.6%)	155 (47.3%)	
**Strong reasoning**						0.004^a^
Agree	48 (58.5%)	42 (51.9%)	26 (31.7%)	35 (42.2%)	151 (46.0%)	
Else	34 (41.5%)	39 (48.1%)	56 (68.3%)	48 (57.8%)	177 (54.0%)	
**Likelihood of use**						
**Likelihood of use**						0.595^a^
Agree	35 (42.7%)	38 (46.9%)	30 (36.6%)	33 (40.2%)	136 (41.6%)	
Else	47 (57.3%)	43 (53.1%)	52 (63.4%)	49 (59.8%)	(58.4%)	

^a^Pearson's Chi-squared test.

Overall, policymakers were similarly likely to use the policy briefs, regardless of the brief type they were shown (Table 4). While the importance of evidence of scientific effectiveness in determining what issues to work on was significantly associated with increased odds of using the policy brief in model 2 (OR = 2.61, 95% CI: 1.40, 5.08), this association was no longer statistically significant after adjusting for the factor created to represent understanding/credibility (aOR = 1.84, 95% CI: 0.89, 3.91). In model 3, policymakers were much more likely to use the briefs if the presence of data on impact in local area or community was important or very important in determining what issues to work on (aOR = 7.39, 95% CI: 1.86, 52.57). Finally, the policy brief understandability/credibility factor score was associated with increased odds of using the policy brief. Specifically, with each change in one standard deviation of the factor, the odds of using the policy brief increased 4.5 times (aOR = 4.72, 95% CI: 3.16, 7.40).

**Table 4:** Logistic regression models predicting likelihood of policy brief use by local policymakers, *N* = 331.

	**Model 1: study design, weighted**			**Model 2: study design** + **policymaker characteristics, weighted**			**Model 3: study design** + **policymaker characteristics** + **brief understandability/credibility factor score, weighted**		
**Predictors**	**OR**	**95% CI**	***p*** **-value**	**OR**	**95% CI**	***p*** **-value**	**OR**	**95% CI**	***p*** **-value**
Brief shown (Framing)	1.19	0.63–2.26	0.595	1.37	0.71–2.67	0.354	1.73	0.82–3.71	0.155
Brief shown (Narrative)	0.64	0.33–1.24	0.189	0.67	0.34–1.33	0.251	0.72	0.32–1.61	0.420
Brief shown (Mixed)	0.90	0.48–1.70	0.755	1.17	0.61–2.27	0.638	1.44	0.67–3.12	0.354
Data on impact in local area/community important in determining policymaker agenda				8.82	2.43–59.81	0.005	7.39	1.86–52.57	0.014
Evidence of scientific effectiveness important in determining policymaker agenda				2.61	1.40–5.08	0.003	1.84	0.89–3.91	0.105
Policymaker rating of brief understandability/credibility factor score							4.72	3.16–7.40	< 0.001
Observations	327			325			323		
R^2^ Tjur	0.008			0.071			0.306		

## Discussion

### Factors influencing what policymakers work on

This study offers several insights into the factors influencing local policymakers' decisions about what issues to work on, their opinions about various formats of policy briefs, and the likelihood that they might use the information provided in the briefs they were shown. Previous work has shown that policymakers at the state level may make decisions about what issues to work on based on constituent needs and opinions and evidence of scientific effectiveness (35). In the current study, local policymakers also indicated that these factors are important in determining their legislative priorities. This should prompt public health practitioners and researchers to help inform constituents about public health issues and potential policy solutions. Further, these findings support the proactive inclusion of scientific evidence in communications with local policymakers.

Most local policymakers in this study also reported that data on the impact of issues in their local area were important or very important in determining what issues they work on. This confirms similar findings suggesting that many legislators prefer local data, and policymaking may be more successful when local data are utilized (3, 36). Fortunately, including or highlighting local data is a strategy researchers, practitioners, and advocates can increasingly implement into communication efforts with relative ease. The availability of local data has improved in recent years and many online resources now exist where these data can be found. Notable examples include the County Health Rankings & Roadmaps (37), PLACES: Local Data for Better Health (38), City Health Dashboard (39), and PolicyMap (40). Also, local data need not necessarily be health-focused. Policy communications materials can also incorporate local data from other sectors relevant to health and effective at engaging policy audiences (e.g., transportation, housing, zoning data, etc.).

A majority of local policymakers in this study also indicated that cost-effectiveness data or economic analysis was important or very important in determining what issues they work on. Legislators face myriad needs and requests to address with a limited budget. Providing them with data showing economic evidence of the burden of health issues or the cost savings of evidence-based interventions may persuade them to work on those issues or encourage their support for an EBP. In a recent study conducted with state legislators, Purtle and colleagues found that including local, economic evidence increased legislator interaction with evidence-based dissemination materials, albeit only among Democratic legislators (41).

These findings support the use of local data, cost or economic analysis, and evidence of scientific effectiveness when communicating with local policymakers. However, the importance of knowing one's target audience and tailoring materials to their interests, political persuasions, and priorities is also crucial, as a “one-size-fits-all” approach may be less effective (3). Previous studies of dissemination to policymakers affirm the importance of considering audience characteristics when crafting messages and determining the format of communication (e.g., policy briefs, social media, video, etc.) (9, 42). Further, in a recent study of state legislators, Smith and colleagues used latent class analysis to identify four groups of policymakers based on their prioritization of various research characteristics and then determined group preferences for receiving information (12). For example, they found that “pragmatic consumers” prefer concise communication, including cost data, while “constituent-oriented decision makers” seek information relevant to constituents and delivered by a trusted source. Their findings highlight the importance of considering the unique values, priorities, and preferences in the development of dissemination materials for policymakers (12).

### Comparison of policy brief types

Overall, local policymakers in the current study found the policy briefs to be believable, accurate, able to hold their attention, and easy to understand, regardless of the version of the brief they received. This could indicate that the type or structure of policy briefs is less important than authors ensuring the information is presented in understandable, accurate, and engaging ways. Ample previous research supports these qualities in materials designed for policymakers (10–12). The lack of significant differences in how local policymakers in this study reacted to the different versions of policy briefs shown may also support the importance of tailoring communications. Narrative-focused, risk-framing-focused, and mixed briefs serve different, valuable purposes depending on the intended audience and communication objective. For example, in the current study, local policymakers reported that narrative communication affected them emotionally more than the other types; thus, in early work on an issue, if one's goal is to raise awareness about a topic, incorporating stories into communication materials may be most effective.

Another explanation of the similarities in responses to each policy brief version may be that, due to careful planning, writing, and collaboration with communication experts, each brief type was similarly well-constructed and thus, similarly received by local policymakers. This is not necessarily true among all communications designed for policymakers, as illustrated by a review of obesity-themed policy briefs. In this review, the authors assessed 100 policy briefs that were readily available online. Of those reviewed, the mean length was five pages, 73% included no tables, and the mean Flesch-Kincaid reading level was 13, which is very high (43). Thus, the consistent quality of the various versions of policy briefs used in the current study may have affected the variability in how local policymakers rated them on the variables of interest.

### Likelihood of using the briefs

Logistic regression analyses showed that the type of brief received was not paramount in determining the likelihood of participants indicating they would use the policy briefs. Policymakers placing a high importance on local data as they determine what issues to work on were over seven times more likely to use the policy brief they were shown than those for whom local data were less important in determining legislative priorities. This finding is significant and actionable, strongly supporting the guidance to those communicating with local policy audiences that local data be an important component in communication materials.

It is important to acknowledge the challenges that may exist in smaller, local organizations seeking to incorporate these suggestions into communications efforts. Many may lack capacity and adequate staff to locate, understand, or utilize local data, even when it is available (44). However, efforts to make materials understandable and credible create opportunities for participatory partner engagement. Identifying other organizations, local college or university staff, local health departments, or even community members who can assist with data location and interpretation can support efforts to create effective policy communications.

Logistic regression analyses also showed an association between the likelihood of using the policy brief shown and finding the policy briefs to be understandable, credible, etc. The higher participants rated the briefs, the greater their chance of indicating they would use them. This finding has high face validity and highlights the value of creating communication materials for local policymakers that are understandable, credible, believable, accurate, and engaging. Doing this requires a study of the target audience as well as utilization of best practices for creating concise, clear messages and including local data (regarding both how an issue influences a local community and the local impacts of policy actions), visual aids (images, icons), and evidence of scientific effectiveness.

Some study limitations warrant mention. While the response rate was typical of other studies with similar populations, it limited the study's statistical power (32, 33). However, the non-response was evenly distributed across demographic variables, likely neutralizing the effect of any bias. Also, respondents' answers to some questions may have been influenced by the social desirability of specific responses. Respondents who value research may be more likely to participate in research, which could introduce bias. Generalizability may be limited by the homogeneity of the sample. It is not possible to determine with certainty what influences policymakers' decisions about what to work on when personal interest, interest groups, constituent opinions, etc., compete for top priority. The issue of timing may also be important, especially in its effect on policymakers' likelihood of using the policy briefs shown. If local policymakers were working on issues related to zoning, housing, or obesity at the time they received the policy brief, they may have indicated an increased likelihood of using the briefs than others who may not have been working on such issues. Finally, as noted, all four brief types were well constructed. This may have diminished the potential effects of the differences among brief types and how they were rated for understandability, believability, etc.

Evidence-based policies can improve public health and help reduce the disproportionate burden of obesity in the United States (45, 46). Getting evidence into the hands of local policymakers can be challenging; further, communicating evidence in ways that make it understandable, credible, and likely to be used requires applying existing knowledge of best practices for sharing information with policy audiences. While various communication objectives may call for different elements in materials designed for policymakers, every effort should be made to incorporate data specific to a policymaker's local area to ensure maximum impact. If researchers, practitioners, and advocates can create policy briefs likely to be used, research is more likely to influence policy.

## Data availability statement

The raw data supporting the conclusions of this article will be made available by the authors, without undue reservation.

## Ethics statement

The studies involving human participants were reviewed and approved by the Washington University in St. Louis Institutional Review Board. Written informed consent for participation was not required for this study in accordance with the national legislation and the institutional requirements.

## Author contributions

RJ performed the statistical analysis. ED wrote the manuscript and all authors contributed to manuscript revisions. All authors contributed to conception and design of the study. All authors contributed to the article and approved the submitted version.
